# Diagnostic Utility of Pleural Fluid Cell Block versus Pleural Biopsy Collected by Flex-Rigid Pleuroscopy for Malignant Pleural Disease: A Single Center Retrospective Analysis

**DOI:** 10.1371/journal.pone.0167186

**Published:** 2016-11-23

**Authors:** Shion Miyoshi, Shinji Sasada, Takehiro Izumo, Yuji Matsumoto, Takaaki Tsuchida

**Affiliations:** 1 Department of Endoscopy, Respiratory Endoscopy Division, National Cancer Center Hospital, Chuo-Ku, Tokyo, Japan; 2 Department of Respiratory Medicine, Toho University Omori Medical Center, Ota-Ku, Tokyo, Japan; 3 Department of Respiratory Medicine, Tokyo Saiseikai Central Hospital, Minato-Ku, Tokyo, Japan; Baylor College of Medicine, UNITED STATES

## Abstract

**Background:**

Some trials recently demonstrated the benefit of targeted treatment for malignant disease; therefore, adequate tissues are needed to detect the targeted gene. Pleural biopsy using flex-rigid pleuroscopy and pleural effusion cell block analysis are both useful for diagnosis of malignancy and obtaining adequate samples. The purpose of our study was to compare the diagnostic utility between the two methods among patients with malignant pleural disease with effusion.

**Methods:**

Data from patients who underwent flex-rigid pleuroscopy for diagnosis of pleural effusion suspicious for malignancy at the National Cancer Center Hospital, Japan between April 2011 and June 2014 were retrospectively reviewed. All procedures were performed under local anesthesia. At least 150 mL of pleural fluid was collected by pleuroscopy, followed by pleural biopsies from the abnormal site.

**Results:**

Thirty-five patients who were finally diagnosed as malignant pleural disease were included in this study. Final diagnoses of malignancy were 24 adenocarcinoma, 1 combined adeno-small cell carcinoma, and 7 malignant pleural mesothelioma (MPM), and 3 metastatic breast cancer. The diagnostic yield was significantly higher by pleural biopsy than by cell block [94.2% (33/35) vs. 71.4% (25/35); p = 0.008]. All patients with positive results on cell block also had positive results on pleural biopsy. Eight patients with negative results on cell block had positive results on pleural biopsy (lung adenocarcinoma in 4, sarcomatoid MPM in 3, and metastatic breast cancer in 1). Two patients with negative results on both cell block and pleural biopsy were diagnosed was sarcomatoid MPM by computed tomography-guided needle biopsy and epithelioid MPM by autopsy.

**Conclusion:**

Pleural biopsy using flex-rigid pleuroscopy was efficient in the diagnosis of malignant pleural diseases. Flex-rigid pleuroscopy with pleural biopsy and pleural effusion cell block analysis should be considered as the initial diagnostic approach for malignant pleural diseases presenting with effusion.

## Introduction

Although pleural effusion is one of the clinical signs of malignant disease, its accurate diagnosis is sometimes difficult. Determining the diagnosis of pleural effusion is important in planning the appropriate management and in the prognostication of the malignant disease [1–3]. Thoracentesis and/or closed pleural biopsy are generally considered as the first step for diagnosis of pleural effusion because these procedures can be easily performed even in outpatients. Some studies have reported that the diagnostic yield of cytology by thoracentesis was 62% to 90% and that of closed pleural biopsy was 40% to 75% [3]. If these procedures turn out to be non-diagnostic, further examination is needed for a definitive diagnosis.

Medical thoracoscopy is a well-established diagnostic procedure for patients with suspected malignant pleural effusion. Earlier studies reported the diagnostic utility and safety of using rigid thoracoscopy [4, 5]. But rigid thoracoscopy is unfamiliar for most pulmonologist because of the technical difficulties, and it sometimes may provide insufficient field of view in the chest wall and need a second entry point. Recently, flex-rigid pleuroscopy under local anesthesia was developed to augment some of the inadequacies of rigid thoracoscopy in evaluating pleural effusion [6–8].

On the other hand, cell block is also a useful method to evaluate pleural effusion by enabling observation of tissue architecture and providing additional sections that are easily available for special stains and immunochemistry [9, 10]. Because of its safe and easy collection, pleural fluid cell block is considered an alternative to pleural tissue, especially if the patient ineligible for surgery or biopsy.

Although pleural biopsy and pleural effusion cell block are both useful for the diagnosis of malignancy, there have been no studies that compared the diagnostic utility between pleural biopsy and the corresponding pleural effusion. Therefore, it remains unclear whether pleural effusion cell block is a useful diagnostic alternative to pleural biopsy for malignancy. The purpose of our study was to compare the diagnostic performance between pleural fluid cell block and pleural biopsy in patients with malignant pleural disease presenting with effusion.

## Materials and Methods

### Patients

This study was a single-center retrospective study to compare the diagnostic yield between pleural effusion cell block and pleural biopsy obtained by flex-rigid pleuroscopy for malignant pleural disease with effusion. Sixty-eight patients who underwent flex-rigid pleuroscopy at the National Cancer Center Hospital, Japan between April 2011 and June 2014 were eligible for this study. Among 68 patients, pleural effusion cell block was prepared in 39 patients; 35 of these patients who were finally diagnosed with malignant pleural disease were included in this study. The remaining 4 patients were diagnosed with benign disease (fibrous pleuritis in 3 and tuberculous pleuritis in 1).

All patients underwent computed tomography (CT) scan of the chest before the procedure. We excluded patients with coagulation disorder, unstable cardiopulmonary dysfunction, persistent hypoxemia, and severe pleural adhesion. National Cancer Center Institutional Review Board approval was granted for this study (No. 2012–278). Written informed consent was obtained from all patients before the procedures.

### Procedure

All procedures were performed by one expert pulmonologist and two assistants. A single puncture technique was used for pleuroscopy, and linear-type ultrasonographic probe was used to determine the appropriate entry site. Patients were positioned in the lateral decubitus position, with the affected side up. Pre-medications included 0.5 mg atropine and 25 mg hydroxyzine hydrochloride, which were administered intramuscularly, and 15 mg pentazocine, which was administered intravenously. Patients received intravenous conscious sedation with midazolam. Arterial oxygen saturation and heart rate were monitored during the examination. Topical anesthesia with 1% lidocaine was administered by infiltrating the skin, subcutaneous tissue, and muscle, and pleural fluid was confirmed with aspiration. After performing skin incision and blunt dissection of the parietal pleura, a flexible trocar with 8 mm inner diameter was inserted into the fifth to the seventh intercostal space at the middle axillary line.

The flex-rigid pleuroscope (LTF-260; Olympus, Tokyo, Japan) was introduced through the trocar; after collecting at least 150 ml of pleural fluid for examination, the rest of the fluid was drained. Thereafter, the entire chest cavity was observed, followed by subpleural injection of saline containing 0.5% lidocaine and 0.005% epinephrine for local anesthesia. Next, multiple biopsies from abnormal areas on the parietal pleura were obtained using flexible forceps (FB-55CR-1; Olympus, Tokyo, Japan). After withdrawing the thoracoscope from the trocar, a 20- to 24-Fr indwelling chest tube was placed through the incision site and connected to a drainage system.

### Biopsy specimen and cell block

Biopsy specimens were immediately fixed in formalin and processed to paraffin blocks and sections. Paraffin embedded sections were Hematoxylin and Eosin (HE) and immunohistochemical stained.

We collected at least 150 mL of pleural fluid into test tubes containing heparin. Specimens were centrifuged for 5 minutes at 1500 rpms. The sediment was stained with the Papanicolaou and Giemsa method. The remaining sediment from the cell block sample was mixed with 20% buffered formalin (pH 7.0) then underwent centrifugation for 5 minutes at 1500 rpms in a pipette tip plugged by welding at the tip. The cell pellets were dehydrated and embedded in paraffin. Finally, paraffin blocks were cut into 3-μm sections for HE staining and immunohistochemistry (IHC). The cytology and pathology results were reported by pathologist.

### Statistical analysis

Data were presented as median (range), frequencies, and percentages. McNemar χ2 statistic was used to compare categorical variables. Statistical analysis was carried out using IBM SPSS Statistics (version 22, IBM SPSS Inc., Chicago, IL). A *P* value of less than 0.05 was considered statistically significant.

## Results

The demographics of the study population are shown in Table 1. Among the 35 patients, 20 (57.1%) were men and 15 (42.9%) were women, with a median age of 66 years (range, 41–81 years). Twenty-nine patients (82.9%) had thickened pleura and 17 (49.6%) had pleural nodules on CT scan. Five patients (14.3%) had a prior history of asbestos exposure. Thirty-one patients underwent flex-rigid pleuroscopy as the initial diagnostic procedure without preceding thoracentesis. Among the remaining four patients who underwent thoracentesis for assessment by the cell block prior to flex-rigid pleuroscopy, diagnosis by cell block assessment included adenocarcinoma, atypical cells, and no tumor in 1, 1, and 2 patients, respectively. On pleuroscopic examination, loculations were found in 10 patients. Median procedure time was 54 minutes (range, 20–107 minutes). Twenty-two patients underwent pleurodesis before chest tube removal. Chest tube drainage was continued for a median duration of 8 days (range, 4–24 days) in patients with pleurodesis and 4 days (range, 1–11 days) in those without pleurodesis. Final diagnoses of malignancy were 24 adenocarcinoma, 1 combined adeno-small cell carcinoma, 7 malignant pleural mesothelioma (MPM), and 3 metastatic breast cancer. One patient developed prolonged pneumothorax that was improved by prolonged chest-tube drainage.

**Table 1:** Demographics of patients with malignant pleural disease (N = 35)

Variables	N (%)
**Median age (years)**	66 (range, 41–56)
**Sex**	
Male	20 (57.1)
Female	15 (42.9)
**Smoking history**	
Current and former	22 (62.9)
Never	12 (34.2)
Unknown	1 (2.9)
**Asbestos exposure**	5 (14.3)
**CT findings**	
Pleural thickening	29 (82.9)
Pleural nodule	17 (49.6)
**Performance status**	
ECOG 0	4 (11.4)
ECOG 1	27 (77.1)
ECOG 2	4 (11.4)
ECOG 3–4	0 (0)
**Pleurodesis**	22 (62.9)
**Loculation on pleuroscopic examination**	10 (28.6)
**Thoracentesis with cell block preceding**	4 (11.4)
**Median procedure time (min)**	52 (range, 20–107)
**Median chest tube drainage period**	
Without pleurodesis (days)	4 (range, 1–11)
With pleurodesis (days)	8 (range, 4–24)
**Median duration from the flex-rigid pleuroscopy to diagnosis** ^**a**^ **(days)**	7 (range, 1–19)

CT, computed tomography; ECOG, Eastern Cooperative Oncology Group

^a^Two patients who could not be definitively diagnosed by flex-rigid pleuroscopy were excluded.

Table 2 shows the comparison of diagnostic yield between cell block and pleural biopsy. The diagnostic yield was significantly higher by pleural biopsy than by cell block [94.2% (33/35) vs. 71.4% (25/35); p = 0.008]. All patients with positive results on cell block had positive results on pleural biopsy. Table 3 shows the diagnostic comparison between cell block and pleural biopsy in 10 patients with negative results on cell block; 8 of these patients had positive results on pleural biopsy (lung adenocarcinoma in 4, sarcomatoid MPM in 3, and metastatic breast cancer in 1). Two patients with negative results on both cell block and pleural biopsy were diagnosed as sarcomatoid MPM by CT-guided needle biopsy and epithelioid MPM by autopsy.

**Table 2:** Diagnostic yield of flex-rigid pleuroscopy according to histology (N = 35)

Final diagnosis	N	Cell block	Pleural biopsy	*P* value^a^
Lung ADC	24	20/24 (83.3%)	24/24 (100%)	0.125
Lung adeno-small combined	1	1/1 (100%)	1/1 (100%)	1.000
MPM	7	2/7 (28.6%)	5/7 (71.4%)	0.250
Breast cancer	3	2/3 (66.7%)	3/3 (100%)	1.000
Total	35	25/35 (71.4%)	33/35 (94.2%)	0.008

ADC, adenocarcinoma; MPM, malignant pleural mesothelioma

^a^*P* values were calculated using McNemar χ2 statistic.

**Table 3:** Comparison of diagnostic yield between cell block and pleural biopsy in patients with negative results on cell block (N = 10)

Patient No	Age	Sex	Diagnosis with cell block	Diagnosis with pleural biopsy	Final diagnosis
1	63	M	no tumor	Success	Lung ADC
2	51	M	no tumor	Success	Lung ADC
3	44	M	no tumor	Success	Lung ADC
4	65	M	atypical cells	Success	Lung ADC
5	69	M	no tumor	Success	Sarcomatoid MPM
6	74	M	atypical cells	Success	Sarcomatoid MPM
7	75	M	atypical cells	Success	Sarcomatoid MPM
8	77	M	no tumor	Failure	Sarcomatoid MPM
9	71	M	Mesothelial cell proliferation	Failure	Epithelioid MPM
10	71	F	atypical cells	Success	Breast cancer

M, male; F, female; ADC, adeno carcinoma; MPM, malignant pleural mesothelioma

## Discussion

Flex-rigid pleuroscopy under the local anesthesia is a valuable procedure to obtain pleural tissue samples and diagnose malignant disease, regardless of the presence of pleural effusion [11–15]. Flex-rigid pleuroscopy offers several benefits that rigid thoracoscopy cannot achieve, including well tolerability, easy handling, superior field of view in the chest wall due to the flexible tip. In addition, it can be performed under local anesthesia [7, 8, 11]. Compared with rigid thoracoscopy, postoperative pain is less likely to be a concern due to the flexible nature of both the trocar and scope. However, size of the biopsy specimens obtained by flex-rigid pleuroscopy is smaller than that collected by rigid thoracoscopy. Although one patient in the present study developed prolonged pneumothorax as a complication, it resolved with prolonged chest-tube drainage; there were no serious adverse events in any of the patients. Subsequently, 22 patients underwent pleurodesis before chest tube removal. Flex-rigid pleuroscopy was well tolerated and should be considered as an initial diagnostic procedure for definitive diagnosis of malignant pleural disease.

The sensitivity and specificity of pleural biopsy using flex-rigid pleuroscopy for exudative pleural effusion have been reported to be 91% and 100%, respectively [11]. Tissue biopsy followed by histologic review is the gold standard for diagnosis, but is not always available. Pleural effusion cell block is a useful alternative because collection is easy and better morphologic preservation of the architectural pattern may be obtained, compared with conventional cytology [16]. In previous studies, the sensitivity of cell block varied widely from 60% to 89.4% [9, 17–20], probably because of differences in sampling type, size, type of specimens, and aspiration techniques. Because it is easier, collection of pleural effusion for cell block samples by thoracentesis generally precedes pleural biopsy by thoracoscopy. However, if cell block is non-diagnostic, treatment would be further delayed. Considering this issue, thoracoscopic pleural biopsy should probably be considered as the first procedure. However, to the best of our knowledge, there have been no reports showing that thoracoscopic pleural biopsy has better diagnostic utility than pleural effusion cell block and that pleural effusion cell block can be a suitable substitute for pleural biopsy for diagnosis of malignancy.

The results of this study showed that pleural biopsy using flex-rigid pleuroscopy had a significantly higher diagnostic yield (94.2%) than pleural effusion cell block (71.4%) for malignant pleural disease. In patients with pleural malignancy, flex-rigid pleuroscopy with pleural biopsy may be a better diagnostic method than thoracentesis alone. In previous studies, pleural biopsy using flex-rigid pleuroscopy was performed after a less invasive procedure failed to yield a diagnosis. In these studies, diagnostic outcomes favored pleural biopsy only in patients with negative cell block analysis, suggesting the presence of bias in patient selection. However, in this study, cell block assessment with thoracentesis did not precede pleural biopsy in 31 out of 35 patients, who underwent flex-rigid pleuroscopy as the initial diagnostic procedure, which should limit selection bias in our study.

**Fig 1:**
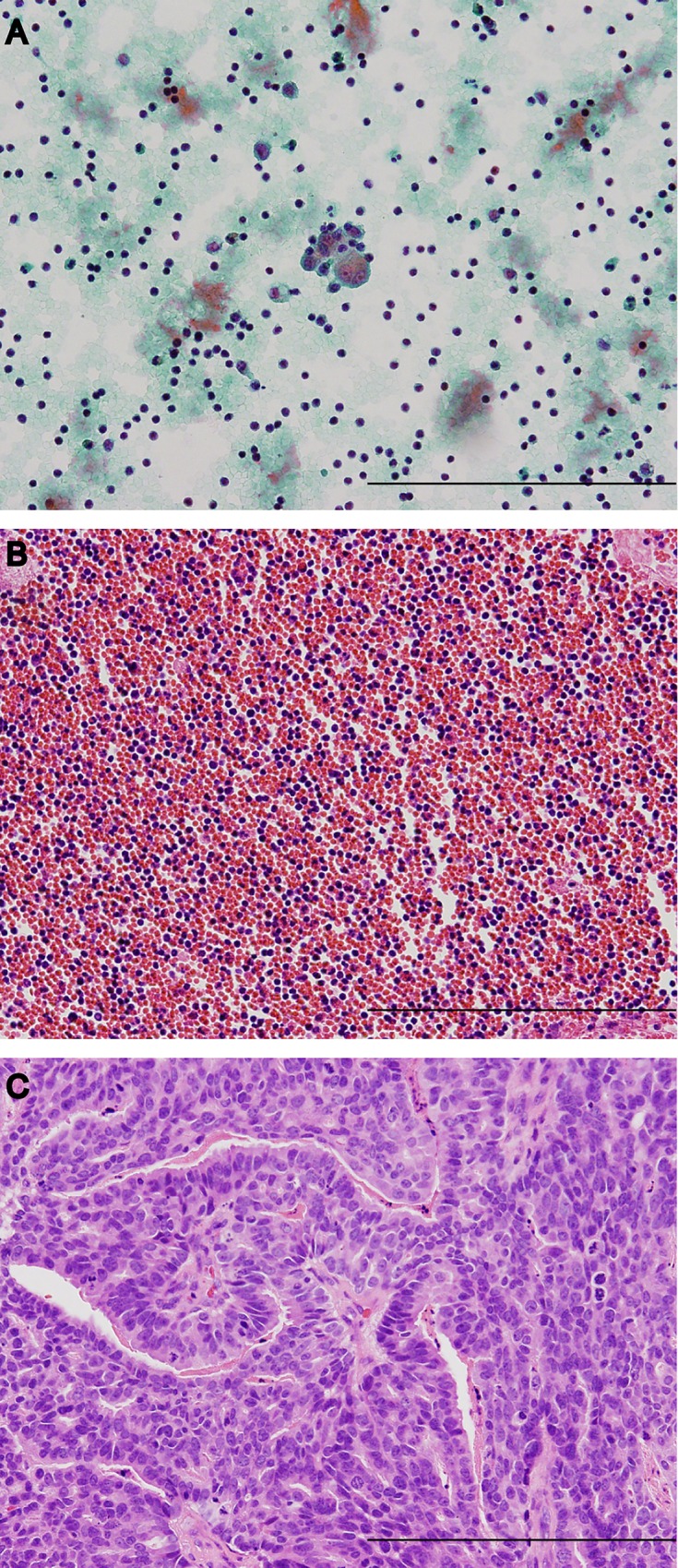
Cytology, cell block and biopsy specimen findings in a patient with negative result on cell block. In 63 years old male, conventional cytology shows few malignant cells with features of adenocarcinoma (Papanicolaou stain) (A). Cell block shows only red blood cells and inflammatory cells with no malignant cells (Hematoxylin and Eosin stain) (B). Biopsy specimen shows adenocarcinoma with solid growth pattern (Hematoxylin and Eosin stain) (C). Scale bar = 200 μm.

Pleural biopsy and collection of pleural fluid for cell block were performed in one procedure during flex-rigid pleuroscopy. In 8 patients with negative results on cell block, pleural biopsy was able to contribute to rapid diagnosis. This low yield of diagnostic malignant cells on cell block may be due to hypocellularity and bleeding in the cell block preparations (Fig 1). The amount of malignant cells was few, as seen in conventional cytology, and this may have contributed to the negative cell block results. Cellularity of biological fluid samples with excessive blood decrease the quality and compromise accurate assessment of specimens [21, 22]. Although not used in the present study, in such cases, treatment with a hemolytic agent is recommended to improve the cellularity in pleural fluid samples with excessive blood [21].

Thoracoscopy is recommended to obtain a sufficient amount of pleural tissue, especially for the diagnosis of MPM [23, 24]. The diagnostic yield of thoracoscopic biopsy has been reported to be >90% [25, 26]. In this study, seven patients were diagnosed as MPM, which consisted of 2 epithelioid type, 4 sarcomatoid type, and 1 biphasic type. All sarcomatoid MPM patients had negative results on cell block, whereas epithelioid and biphasic MPM patients had positive results on cell block. These results suggested that pleural biopsy using flex-rigid pleuroscopy may be an especially useful procedure when sarcomatoid MPM is suspected [27]. The sensitivity of cytological diagnosis of mesothelioma has been reported to range from 32% to 76% [28–30]. Although the sensitivity may be related to sampling and experience of cytopathologists, the diagnosis of epithelioid MPM can be established based on effusion cytology. However, it is generally considered that malignant cells, especially in sarcomatoid MPM, do not float in the pleural fluid because of the overlying reactive epithelioid mesothelial cells and the tight organization of tumor tissue [28]; therefore, pleural effusion cell block seems to be not useful for the diagnosis of sarcomatoid MPM. Our findings were consistent with this observation. Thus, pleural biopsies should be obtained under visual guidance in patients with suspicious sarcomatoid MPM.

Malignant cells are considered to be present heterogeneously within the pleural effusion [31] and can be precipitated by gravity. Position of the patient’s body and the site of puncture may affect the diagnostic yield of cytology or cell block from thoracentesis. In our study, this heterogeneity of pleural fluid cells probably did not affect the diagnostic yield of the cell block preparations because pleural fluid was collected under direct vision by flex-rigid pleuroscope. However, scarcity of free malignant cells in pleural fluid samples remains a serious challenge. A prospective study demonstrated that at least 150 mL of pleural fluid is needed for analysis with both cytology and cell block [32]. Consequently, ≥150 mL pleural was routinely collected in all patients in the present study. However, the volume of pleural fluid can be increased if assessment by cell blocks requires more cell volume.

Pleural loculations on pleuroscopic examination were observed in 10 patients in the present study. Loculated and free-flowing pleural effusion exhibit many distinct features that are clinically important [33], which may impact the subsequent analysis of pleural fluid samples collected from different locules [34]. In the present study, cell blocks were non-diagnostic in 5 out of the 10 patients with pleural loculations and in 5 out of 25 patients without pleural loculations. The rate of non-diagnosis tended to be higher in patients with pleural loculations than in those without loculations, demonstrating the relationship between the presence of loculations and negative results with cell block analysis. Therefore, it is essential to evaluate every undiagnosed pleural effusion by routine ultrasound examination to obtain additional information such as the presence of loculations.

Targeted treatment has been developed for malignant diseases, such as non-small cell lung cancer with epidermal growth factor receptor (*EGFR*) mutation [35, 36] and anaplastic lymphoma kinase (*ALK*) [37], and breast cancer with human epidermal growth factor receptor 2 [38, 39]. Some trials demonstrated the benefit of such targeted treatments; therefore, adequate tissues are preferred in order to detect the targeted gene by IHC or fluorescence *in-situ* hybridization (FISH). When tissue biopsy is not available, malignant pleural effusion sample may be used for these analyses. FISH using fresh pleural fluid samples should be considered as an additional informative assay for pleural effusions suspicious of being malignant [40, 41]. Furthermore, cell block preparations are commonly used for diagnostic pathology and gene analysis by FISH and IHC [42, 43]. A few reports reported that compared with tumor tissue, pleural effusion cell block had 81.8% sensitivity and 80% specificity for detection of *EGFR* mutation [44] and 62.5% to 100% sensitivity and 100% specificity for *ALK* detection [43]. For successful detection of the targeted gene, sufficient amount of malignant cells are needed; however, in some cases, like our patient (Fig 1), the amount of malignant cells in cell block samples are few. In such cases, tumor tissue is considered more suitable than cell block for targeted gene detection. Moreover, third generation *EGFR* inhibitors have been developed to overcome a second *EGFR* mutation, T790M, which reduces binding of first generation *EGFR* inhibitors [45]. Re-biopsy of patients with acquired resistance is required in order to guide the choice of second-line therapies. In a previous study, the diagnostic rate for T790M mutation by lung biopsies and pleural fluid samples were 67% and 37%, respectively [46]. Additionally, the heterogeneity of T790M-positive cells within pleural fluid samples were reported [47]. Thus, tissue samples are likely to have higher detection rate than pleural fluid samples for T790M mutation. However, there are currently no data on the concordance rate between pleural tissue and matched pleural fluid samples for T790M detection.

Recently, the usefulness of non-invasive detection of targeted gene in plasma samples has been reported. T790M mutation in plasma samples was detected by circulating tumor cells or circulating tumor DNA, with a concordance rate of 57% to 83% between tissue and plasma samples [48, 49]. This discordance is thought to result from heterogeneity of tumor samples and technical differences. Nevertheless, these two samples are thought to complement to each other in terms of targeted gene detection, especially T790M mutation. To date, gene analysis on fluid samples has been established based on correlation with tissue samples. Therefore, accurate diagnosis and targeted gene detection by adequate tissue sampling remain to be important in validating fluid sample analysis.

In the current study, Pleural biopsy using flex-rigid pleuroscopy had a better diagnostic yield than pleural effusion cell block assessment for the diagnosis of malignant pleural diseases. However, these results do not implicate pleural biopsy alone as sufficient for diagnosis. There is a possibility that the tight organization of pleura or other unpredictable complication such as major bleeding can lead to failure of pleural biopsy. Pleural fluid should be collected at the time of flex-rigid pleuroscopy as an easy, safety and useful diagnostic approach.

There were several limitations of our study. First, the study was performed in a single cancer center, leading to a possible bias in patient selection. Second, this was a retrospective analysis of a small sample size population, in which the patients were finally diagnosed as malignancy. A randomized, multi-center trial is needed to validate these findings.

## Conclusion

Pleural biopsy using flex-rigid pleuroscopy was efficient in the diagnosis of malignant pleural diseases. Flex-rigid pleuroscopy with pleural biopsy and pleural effusion cell block analysis should be considered as the initial diagnostic procedure for malignant pleural diseases presenting with effusion.
